# Understanding physician burnout in Oman: current status, cultural influences and future directions

**DOI:** 10.1192/bji.2024.1

**Published:** 2024-05

**Authors:** Mohammed Al Alawi, Abdullah Al Ghailani

**Affiliations:** 1 MD, MRCPsych, Consultant Liaison Psychiatrist, Department of Behavioral Medicine, Sultan Qaboos University, Al-khod, Oman.; 2 MD, Psychiatry Resident, Oman Medical Specialty Board, Al-Athaiba, Oman.

**Keywords:** Resident burnout, cross-cultural, medical students, stress, prevalence

## Abstract

Amidst the current global surge in physician burnout, a compelling need arises for precisely targeted research and interventions that cater to specific contexts, illuminating a path towards professional well-being. This brief communication analyses recent studies on physician burnout in Oman, critically evaluating the findings, cultural factors, methodological limitations and future growth opportunities. Distinct elements of Omani culture, encompassing attitudes towards mental illness, gender roles and patient expectations, can distinctly influence how burnout presents in this population. Advanced mixed-methods research integrating cultural insights, biomarkers and longitudinal tracking is needed to characterise burnout in Omani physicians. The findings can play a significant role in developing comprehensive interventions, at both a systemic and an individual level, that promote well-being of physicians while specifically aligning with the cultural values of Oman.

Burnout – characterised by emotional exhaustion, depersonalisation and reduced personal accomplishment^1^ – is increasingly prevalent among physicians internationally.^2^ Consequences span diminished quality of life, suboptimal patient care and workforce attrition.^3^ Although extensive research exists, critical evaluation reveals major limitations, including lack of longitudinal designs, over-reliance on the Maslach Burnout Inventory (MBI) and scarce cross-cultural assessments.^4,5^ This brief communication analyses recent empirical studies focused on quantifying and characterising burnout specifically among physicians in Oman, an understudied non-Western cultural context. The investigation navigates through discovered outcomes, cultural impacts, constraints in methodology and prospects for future development. This collaborative exploration seeks to drive significant progress within the domain of research on physician burnout in the context of Oman.

## Method

We adopted a narrative review methodology with a comprehensive search of PubMed and Google Scholar, employing different combinations of key terms such as ‘burnout’, ‘wellbeing’, ‘distress’, ‘physicians’, ‘doctors’, ‘residents’, ‘interns’ and ‘Oman’. Additional relevant articles were identified through the careful scrutiny of reference lists. Emphasis was placed on recent publications that specifically evaluated burnout levels within the Omani physician population, utilising standardised instruments.

## Results

### Prevalence

Recent Omani studies consistently report that burnout peaks during residency rather than in medical school or independent practice.^2,6^Burnout is seen among Omani medical students at a rate of 7.4%, rising in interns to 15%, further rising in residents to 16.6% and affecting practising physicians at a rate of 6.3%.^6,7^ Overall, burnout rates appear lower among Omani compared with Western physicians.

### Risk factors

Potential explanatory factors worldwide for burnout during residency training include high workload, long hours and inadequate preparedness at this early career stage.^8^ Available data suggest that Oman's unique sociocultural dynamics are also likely influencing the way burnout is experienced within this population.^6^ For instance, the strong emphasis on collectivism in Oman might lead to the suppression of personal distress in order to preserve group harmony.^6,7^ The widespread stigma towards mental illness also hinders open acknowledgement of psychological struggles.^6^ Furthermore, traditional gender expectations prescribing emotional strength for men may prevent disclosure of distress. Patients’ cultural beliefs about physicians as infallible healers further compound pressures.^9^ However, quantitative research designs cannot capture the nuances of how these elements influence burnout, and cross-sectional designs using self-report surveys limit causal inferences about potential risk factors.

## Discussion

### Methodological limitations

Most Omani burnout studies employed cross-sectional self-report questionnaires such as the MBI. This allowed identification of predictors such as extended working hours.^6^ However, longitudinal mixed-methods research is needed to elucidate the complex interrelationships between risk factors, burnout dimensions and outcomes. The MBI has also received criticism regarding its validity and overlap with depression.^5^ Developing and validating context-specific burnout measures using qualitative insights and experimental approaches is critical.

### Towards culturally valid assessments

The findings reveal how sociocultural forces uniquely shape burnout manifestations in Oman, necessitating in-depth qualitative exploration of physicians’ experiences. Experimental studies manipulating variables such as social support would further isolate cultural mechanisms. Integrating biomarkers such as cortisol, inflammatory markers, heart rate variability and electroencephalogram abnormalities as objective outcomes might also enhance validity.^10^ Despite limitations, these initial studies lay the foundation for developing robust, culturally calibrated burnout assessments tailored to Omani physicians.

### Future directions

Although burnout is clearly a salient issue among Omani physicians, substantial knowledge gaps persist. Advancing this important research area will require meticulous qualitative inquiry into the cultural shaping of burnout, rigorous longitudinal tracking using culturally adapted metrics and focused measurement studies. The resultant insights could then powerfully inform interventions – spanning individual coping skills to institutional policies – carefully tailored to promote physician well-being within the Omani cultural context.

## Data Availability

The data that support the findings of this study are available on request from the corresponding author, M.A.A.
